# Humans-livestock predators conflict in the Central and Eastern Part of Bale Mountains National Park, Ethiopia

**DOI:** 10.1186/s12862-022-02065-y

**Published:** 2022-10-04

**Authors:** Israel Sebsibe

**Affiliations:** Department of Biology, Salale University, P.O. Box. 245, Fiche, Ethiopia

**Keywords:** Bale Mountains National Park, Livestock-predators, Conflict

## Abstract

**Background:**

Sharing of space by humans and wildlife at a time may ignite apparent conflict. Populations of many species are declining due to the degradation of wildlife habitats caused by agricultural activities. Additionally, livestock may compete with wild herbivores for grazing and reduce the abundance of wild prey for carnivores. A reduction in populations of prey species of large predators might cause carnivores to be attracted towards livestock, ultimately provoking and aggravating the human-carnivores conflict. This study investigated the current status of the human-predators conflict in and around the Bale Mountains National Park.

**Results:**

Most (72.75%) respondents agreed on the presence of livestock predation. Major reported predators were spotted hyena (*Crocuta crocuta*), olive baboon (*Papio anubis*), African wolf (*Canis lupaster*), aardvark (*Orycteropus afer*), genet (*Genetta genetta*), Ethiopian wolf (*Canis simensis*), lion (*Panthera leo*), and leopard (*Panthera pardus*). Cattle (54.19%), sheep (70.96%), goat (32.0%), donkey (37.72%) and horse (27.54%) were mentioned as major target of predators. Within the past ten years 1623 sheep, 741 cattle, 639 goats, 193 donkeys, and 124 horses were predated. This study found an increasing trend of livestock predation. The trend was reported to be high within the Park (68%). During the past ten years, households reported killings of 3320 livestock that cost 347,460.53 USD. Loss of 8.66 USD per month constituted 27.45% of their monthly income which is expected to have a great sustenance impact. Human settlement (41%), agricultural practices (38.6%), overgrazing (25.3%), deforestation for charcoal production (25.1%), deliberate fire to free lands for agriculture (17.3%) were noticed as major causes of livestock depredation.

**Conclusions:**

The results of the present study show that there is strong human-livestock predator conflict in the study area. Therefore, the author suggested that conflict mitigation efforts focus on securing the livestock enclosure to protected areas and regular compensation fees for farmers that face great damage from wildlife. The foremost action should be awareness creation about the environmental, social, and economic importance of protected areas. The management staff of the Park is also expected to promote community involvement in the plan of mitigation strategies and practices.

**Supplementary Information:**

The online version contains supplementary material available at 10.1186/s12862-022-02065-y.

## Introduction

People together with their livestock survive in and around many protected areas in Africa. Due to plenty of natural resources, rural people are always attracted to the abode of many wildlife species. Sharing of habitats by humans and wildlife at a time may ignite clear conflict and one causes an adverse impact upon the other [6]. For instance, various activities on natural habitats may exacerbate human-wildlife conflict by inducing habitat fragmentation through which living space for wildlife gets diminished [38, 43]. Human-wildlife conflict occurs in different forms all over the world. One of the major forms of conflict between local people and wildlife is livestock predation [3]. Interactions between several carnivore species and domestic animals have been very complex for a long time [9]. Predators are rarely a threat to humans in Africa, but they are a significant source of livestock losses to both commercial and subsistence livestock producers [11].

Carnivores including lions, leopards, cheetahs, spotted hyenas, wild dogs, and crocodiles, are frequently noticed as main predators causing a great threat to livestock and responsible for the majority of human-wildlife conflicts. This can impose important economic costs to local communities [42, 47] and the subsequent elimination of predators through retaliatory action is one of the most omnipresent problems faced by carnivores. This is probably due to local people’s view of these large carnivores as government properties and they always want to take measures against them to express their opposition [19].

Populations of many species are declining due to the degradation of wildlife habitats caused by agricultural activities [34, 39]. Additionally, livestock may compete with wild herbivores for grazing and reduce the abundance of wild prey for carnivores [48]. A reduction in populations of prey species of large predators might cause carnivores to be attracted towards livestock, ultimately provoking and aggravating the human-carnivores conflict.

Adverse interactions between humans and carnivores have led to severe results including the extinction of some animal species. According to [21], there is a positive relationship between historical patterns of large carnivore extinction probability and human population density. For example, conflict with people over sheep depredation led to the extinction of two carnivorous mammals, the thylacine or marsupial wolf (*Thylacinus cynocephalus*) in 1930 [48]. Also, the conflict between Ethiopian wolves and pastoralists in different parts of the country has put the species into an endangered state [8].

In the Bale Mountains National Park, different predators are residing, however, they face often conflict as the local people have a tradition of livestock rearing [37]. Livestock production is the best income finding way for many people living in and around the Park. Thousands of people live within the territory of the Park and near the Park boundary (within < 5 km). Due to the lack of natural prey in the Park (needs investigation), predators are turning to livestock to survive. In return pastoralists eventually kill wildlife to protect their livestock. Retaliation measures taken by pastoralists put very important endemic species including the Ethiopian wolf (*Canis simensis*) in an endangered state [8].

However, while Ethiopia has a number of land and other natural resource use policies in place, the government often fails to implement effectively these policies as well as Park management strategies. As mentioned above, thousands of people live inside and near the Park. Government officials and the Park administrators cannot re-locate the people to other places due to budget constraints and people's unwillingness. Mainly pastoralists have been highly dependent on the Park’s resources to sustain their livestock production, turning the Park into grazing land. Due to lack of access to information, most pastoralists are not aware that killing wildlife is illegal and punishable. Furthermore, protecting livestock from predators is acceptable as heroic activity in society since their life depends on them. Since there is no applied compensation strategy for livestock loss from the Park or the government side, people often prefer to use their harmful strategies to protect the livestock. Therefore, it is apparent that the majority of predators and pastoralists are in intense conflict.

Therefore, the present study aims to investigate the human-predators conflict in and around the Bale Mountains National Park. Due to its enormously wide catchment area which covers 247,000 hectares of land with an altitudinal range from 1500 to 4377 m a.s.l, there is a great need for investigation and assessments to find out the overall effect of human and wildlife conflict [10]. Selected sites of the present study have never been assessed to find livestock depredation extent except for Rira. In the present study, major conflict-causing predators and root causes of livestock depredation were identified. The economic loss of livestock depredation of the past decade was also analyzed. Local people’s attitude towards conflict and their mitigation strategies was also assessed.

## Result

### Location and grazing land ownership of the respondents

A greater proportion of respondents live outside the Park boundary (83.8%). From the remaining proportion, 4.93% of respondents live within the Park, 4.07% live near to the Park up to 1 km, and 7.2% live over 5 km outside the Park boundary. Most of the respondents (56.59%) had their private grazing land. The mean size of private grazing land in study sites was 1.15 ± 0.1 ha per household. There was a strong positive correlation between distance from the Park and the private grazing land ownership (r = 0.93, P < 0.05).

### Livestock predation

Most (72.75%) respondents reported the presence of livestock predation. The livestock loss caused by predators was also significantly differed across the study sites (χ^2^ = 16.06, P < 0.05). The degree of the conflict was reported to be more intensive within 1 km and 5 km range from the Park than within the Park (r = 0.76, P < 0.05).

Eight livestock predators were reported in the study area. These predators were spotted hyena (*Crocuta crocuta*), olive baboon (*Papio anubis*), African wolf (*Canis lupaster*), aardvark (*Orycteropus afer*), genet (*Genetta genetta*), Ethiopian wolf (*Canis simensis*), lion (*Panthera leo*), and leopard (*Panthera pardus*). Kebeles were differed in the number of reported predators (F_5 328_ = 3.82, P < 0.05). All predators were mentioned by respondents of Rira who live within territories of the Park (P < 0.05). Tierce (33%) of the respondents reported olive baboons and spotted hyenas as major predators of their livestock. About 28% of respondents reported sightings of African wolves with olive baboons and spotted hyenas. Many who blamed African wolves were from Rira (58.5%) which took 4.5% proportion of all respondents. Some (11.4%) of the respondents mentioned aardvark and genet for chicken depredation. Very few (3.3%) respondents, exclusively from FA (1 km distance from the Park) of and Rira (Within the Park) mentioned Ethiopian wolf. A few people reported that some of the livestock were injured and killed by lions (0.9%) and leopards (1.5%) (Table 1).

**Table 1:** Major livestock predators sighted by respondents in and around BMNP (%)

Predators	AT	WT	FA	WA	IS	Rira	Total
Spotted hyena	2.69	3.89	2.99	0.9	3.3	0	13.77
Olive baboon	2.4	2.99	1.8	1.8	1.2	0	10.19
African wolf	0	0	0	0	0	4.5	4.5
Spotted hyena and olive baboon	14.37	6.59	4.5	7.19	0.3	0	32.95
Spotted hyena, olive baboon and African wolf	6.29	5.09	3	4.79	5.99	2.7	27.86
Aardvark and genet	5.09	2.99	0	2.1	1.2	0	11.38
Ethiopian wolf	0	0	1.5	0	0	1.8	3.3
Lion	0	0	0	0	0	0.9	0.9
Leopard	0	0	0	0	0	1.5	1.5
No predation	4.49	3.59	3.89	4.49	8.38	2.1	26.95

Kebeles: AT-Aloshe Tilo (> 5 km far), WT-Weltai Tosha (> 5 km far), WA-Weltai Azira (> 5 km far), FA-Fassil Angesso (< 1 km close), IS-Ititu Sura (> 1–5 km far), and Rira (within the Park)

There was no correlation between livestock predation and distance from the Park (r = 0.26, P > 0.05). This analysis showed that reported predators do not attack livestock because local people are living in or near the Park. Respondents who faced more livestock predation had negative attitude towards predators (r = − 0.58, P < 0.05). And respondents who faced a few livestock predation events had a positive attitude towards wildlife (r = 6.18, P < 0.05).

Cattle (54.19%), sheep (70.96%), goat (32.0%), donkey (37.72%) and horse (27.54%) were mentioned as major target of predators. Many respondents who live near to the Park (74.6%), far from the Park (61.05%), within the Park (80.5%) reported sheep depredation. A cattle depredation report was provided by 18.3% of respondents. A great proportion of goat depredation was in Rira (73.2%) and FA (52.3%). All the rest of kebeles’ response to goat depredation was under 35%. Much of the pack animals were predated from (51%) far from the Park and the least (19%) from within the Park (Table 2).

**Table 2:** Percentage of livestock predation during the ten years (2009–2018 G.C) in the study area

Depredated livestock						
Kebeles	n	Cattle (%)	Sheep (%)	Goat (%)	Donkey (%)	Horse (%)
AT	80	18.26	19.5	1.8	10.2	6.28
WT	57	9.28	12.3	5.39	8.68	4.79
FA	44	6.28	9.28	6.89	5.09	4.79
WA	48	9.58	9.88	4.19	6.59	5.68
IS	64	6.58	10.2	4.79	3.59	4.49
Rira	41	4.19	9.88	8.98	3.59	1.49
total	334	54.19	70.96	32.0	37.72	27.54

The majority (98.26%) of cattle were depredated by spotted hyenas in the byre at night time and the rest were killed by lions in Rira. Hyenas were reported to attack cattle whether they were in the byre or outside. But the lion did not break byre. Rather it focuses on cattle that stay outside the byre at the night. Many sheep (59.8%) and goats (65.8%) were killed by olive baboon while 27.9% of sheep and 29.2% of goats were killed by African wolves and 7.9% of sheep by spotted hyenas. The Ethiopian wolf was also reported to kill lamb (1.2%) and goats (2.6%) within 1 km distance from the Park and within the Park. Ethiopian wolves did not dare to kill adult sheep. In addition to the Ethiopian wolf (Table 3), leopards were one of the predators of sheep and goats, responsible for the loss of sheep (3.15%) and goats (2.4%).

**Table 3:** Percentage of predators responsible for each species of livestock (%)

Predators	Cattle	Sheep	Goat	Donkey	Horse
Olive baboon	0	59.82	65.8	0	0
Spotted hyena	98.26	7.85	0	98.44	100
African wolf	0	27.97	29.1	0	0
Ethiopian wolf	0	1.19	2.58	0	0
Lion	1.73	0	0	1.55	0
Leopard	0	3.5	2.43	0	0

### The trend of livestock predation

**Fig. 1:**
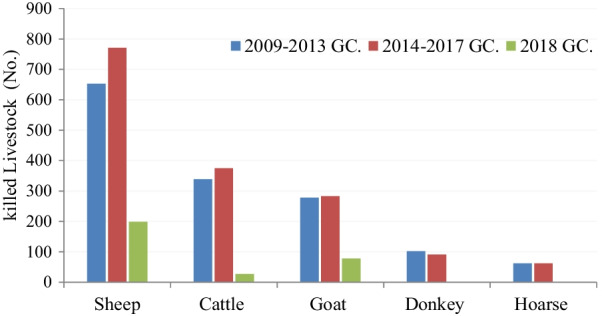
Trend of livestock damage during 2009–2018 GC

All kinds of livestock, 1623 sheep, 741 cattle, 639 goats, 193 donkeys, and 124 horses were killed by predators in all sites from 2009 to 2018 G.C (Fig. 1). During these 10 years, 4.85 ± 0.09 sheep and 2.21 ± 0.05 cattle were depredated per household. Approximately 2 goats were killed from each household (1.91 ± 0.02). The total number of killed livestock in the study area was 3320. The mean number of all depredated livestock within the ten years was 9.94 ± 0.15 per household. There was a significant difference among livestock depredated within the ten years (χ^2^ = 432.2, P < 0.001). The real difference was found when cattle and sheep were compared with other livestock (P < 0.05). Therefore, sheep were highly predated livestock and then cattle. Horse predation was the lowest (Table 2).

All kinds of livestock were depredated in all the sites from 2014 to 2018 G.C (Fig. 1). Totally 970 sheep, 402 cattle, 361 goats, 91 donkeys, and 62 horses were predated (see Additional file 2). There was a significant difference in depredated livestock across kebeles (χ^2^ = 303.6, P < 0.001). Generally, 5.85 ± 0.13 livestock were depredated within the five years per household.

Totally 199 sheep, 27 cattle, and 78 goats were killed by 2018 G.C (Fig. 1). The predation of the three livestock was seemingly reduced this year in many villages, even though it does not mean that the general trend of predation is reduced. And there was an increasing rate of sheep and goat depredation. 1.15 ± 0.02 livestock was lost from each household. There was no reported predation of pack animals during 2018 GC.

There was a significant difference among respondents in their response to the trend of livestock depredation (χ^2^ = 47.68, P < 0.05). Almost half of (49.01%) respondents mentioned the increasing trend of livestock predation. The trend was reported to be high within the Park (68%). Nearly, 17% of respondents considered the trend as constant. Some of the respondents (14.34%) believed livestock depredation is getting reduced. but no one was convinced in Rira that predation is reduced.

### The economic loss of households

The households reported killings of 3320 livestock that cost 347,460.53 USD from 2009 – 2018 G.C. Specifically, 1040.3 USD (~ 1013.8 USD = median) was lost from each household through livestock depredation (Table 4). Farming and small-scale trades are major sources of money for the majority of the respondents from which they earn an average of 31.57 USD per month. Loss of 8.66 USD per month constituted 27.45% of their monthly income which is expected to have a great sustenance impact.

**Table 4:** Total monetary loss of livestock predation in the study sites

Target livestock	Approximate unit cost (USD)	Killed (within 10 years)	Total monetary loss (USD)
Sheep	39.47	1623	64,065.79
Cattle	263.16	741	195,000.00
Goat	52.63	639	33,631.58
Donkey	131.58	193	25,394.73
Hoarse	236.84	124	29,368.42
	Total	3320	347,460.52

### Local predation-curbing techniques

Different predator controlling methods were put into effect for several decades in the study area. Respondents mentioned six major mechanisms they used to control predators. These methods were watch-defend (95.21%), pen construction (67.96%), using dogs (59.28%), direct kill (5.69%), using poisoning (17.2%), and destruction of predators’ habitat (7.49%) setting fire (Table 5). Respondents (17.2%) did use poisons to kill lions and leopards within the Park.

**Table 5:** Preventing mechanisms of predators used by local people in the study area (%)

Methods	AT	WT	FA	WA	IS	Rira	Total
Watch-defend	20.95	16.77	13.17	13.17	19.16	11.98	**95.21**
Pen construction	15.57	14.67	5.69	9.58	16.17	6.29	**67.96**
Using dogs	10.78	14.67	5.69	6.89	16.17	5.09	**59.28**
Direct killing	1.50	0.60	0.00	1.50	0.00	2.10	**5.69**
Poisoning	0.00	0.00	0.00	0.00	0.00	17.2	**17.2**
Destruction of predators habitats	0.00	0.00	1.20	0.00	0.00	6.29	**7.49**

### Causes of livestock predation

**Fig. 2:**
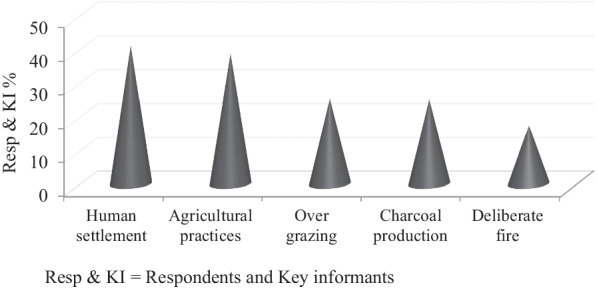
Major causes of livestock predation in the study area

Human settlement (41%), agricultural practices (38.6%), overgrazing (25.3%), deforestation for charcoal production (25.1%), deliberate fire to free lands for agriculture (17.3%) were noticed as major causes of livestock depredation (Fig. 2). Many people living in and around the Park did not have to have their private grazing land. Approximately, 28% of respondents are living within the Park and used it as a grazing field. According to what was reported by the Kebele administration, there was about 25,000 livestock within the Park up to October 2019 GC. A total of 874 ha of the field were used for agriculture in the central part of The Park. Many wild herbivores were noticed (66.3%) to graze outside the Park boundary. This situation favored predators to have double options; one is predating livestock graze within the Park or following the natural preys out of the Park which may alongside expose livestock graze near and far from the Park.

### Suggested mitigation strategies

Different suggestions were given by key informants that are supposed to reduce predation: reduce predators’ number, return them to the Park, compensation for inhabitants of the Park, awareness creation to know how to control predators, and no idea. The former four were what key informants expect the government (the Park management) to do. Some respondents (5.69%) thought that the number of olive baboons and spotted hyena is outstandingly great. The majority of the key informants recommended that killing problematic predators might reduce their effect. But 20% of KIs thought that returning them to the Park is acceptable than killing wildlife. Many (39.2%) of the respondents want to learn some techniques from the government to reduce livestock damage.

## Discussion

The result of the present study revealed that there is a strong human-predator conflict in and around BMNP. However, many respondents had a positive attitude toward wildlife conservation, which did not correlate with their distance from the Park (P > 0.05). Because without going to the Park, they were able to interact with different wild animals found outside the Park. Having a positive attitude towards wildlife conservation has not been a new phenomenon for many African countries. Many studies conducted in Africa revealed that the majority of the local people have a positive attitude towards wildlife conservation. Studies [15, 17, 31] noticed that many respondents had a positive attitude towards wildlife conservation in Zimbabwe, South Africa, and Namibia. As the present study showed, respondents whom predators highly attacked had a negative attitude towards wildlife conservation (r = − 0.64, P < 0.05). A study [36] reported a negative attitude about wildlife conservation among those who faced more attacks from carnivores in Norwegian. A study conducted in Ethiopia [28] also found that respondents who confronted more attacks from carnivores had a negative attitude towards wildlife conservation. According to [13], the positive attitude of local people may be changed due to the high level of conflict. Similarly, conflict mitigation methods are critical to keeping local people’s attitudes positive. The positive attitude of coexisting people is an essential part of carnivores’ fauna conservation and management efforts. Based on the evidence from several studies, including the present, the author suggested that the involvement of the local people in the conservation programs and activities will enhance the implementation of conflict resolution strategies in the Park.

Most (72.75%) respondents agreed on the presence of livestock predation. All mentioned livestock predators were spotted hyena, olive baboon, common jackal, aardvark, genet, Ethiopian wolf, lion, and leopard. Common jackals, leopards, spotted hyenas, and Ethiopian wolves were also found to be livestock predators in different parts of the country [30]. In another part of Africa, baboons, lions, leopards, and hyenas were also mentioned as significant predators responsible for damaging and killing livestock [35]. One-third of respondents reported olive baboons and spotted hyenas as major livestock predators in the study area. Research concerning human-wildlife assessment in Malawi found baboons as crop raiders and predators [4]. One study [3] counted 704 livestock predation in the Web Valley of BMNP (1999–2002). According to the above study, hyenas were responsible for 57% of the livestock predated. In the previous study, leopards were also responsible for goats and sheep depredation. A study conducted in the Menz Guassa district of Ethiopia [12] also reported the loss of sheep, goats, cattle, donkeys, and horses by common jackal, Ethiopian wolves, and hyenas with one additional predator in the Menz Guassa district of Ethiopia. In Kenya, predators such as lions, leopards, and hyenas were responsible for cattle, sheep, and goat losses [11]. Therefore, livestock predators are almost similar throughout the country and the continent.

There was no relationship between the number of livestock predators and distance from the Park (r = − 0.1, P = 0.001). This analysis showed that reported predators attack livestock, not for local people are living in or near the Park. There was a different conclusion given in some other related studies. According to [29], sheep loss to Ethiopian wolves was due to livestock approach to Ethiopian wolf habitat. Several wildlife species may have been comprised within legal boundaries such as parks and protected areas. Nevertheless, the distribution of wildlife in Bale could be very different. However, BMNP harbors essential endemic species such as Mountain nyala and Ethiopian wolf [44]; many other animal species live outside the Park territories, which makes them confront humans and their livestock.

There was an increasing trend of livestock depredation in the study area. The predation trend estimation across Kebeles indicated an increasing rate so that the number of predated livestock is expected to increase in the next decade. Almost half of (49.01%) respondents stressed the increasing trend of livestock depredation. This may be due to increasing trends of expanding grazing lands into the Park territory due to livestock population growth and droughts. With the expectation of many depredation events, local people have been using six techniques for curbing the impact of carnivores’ attacks in the study area. Among all the techniques used, dog rearing, direct killing, poisoning, and destroying habitats of predators can have a devastating impact on the wildlife of the Park. For instance, 17.2% of respondents who live within the Park used a poisonous substance to kill lions. This could affect the ecology and, ultimately, biodiversity by disturbing prey-predator interaction. The local people also setting fire to destruct the habitat of predators to chase them away. Alers et al. [2] and Vial [43] discussed that the causes of fire in BMNP are anthropogenic.

The present study identifies five root causes of livestock predation in and around the Park. Human settlement (41%), agricultural practices (38.6%), overgrazing (25.3%), deforestation for charcoal production (25.1%), and firing wildlife habitats (17.3%) were significant causes of livestock depredation. During the study time, approximately 25,000 livestock grazed within the Park boundary. Furthermore, 874 ha field was already occupied for agricultural purposes inside the Park. A study conducted in another part of the Park found that agricultural expansion, human settlement, overgrazing by livestock, deforestation, illegal grass collection, and poaching are Root causes of human-wildlife conflicts [37]. Another study from the Tsavo Conservation Area, Kenya, reported human settlement, Agricultural Expansion, deforestation, and poaching as the leading causes of Human-wildlife conflicts [22]. Also, life loss and injuries of humans and animals, crop and property damage, agricultural encroachment, developmental activities, and livestock grazing were major reasons for human-wildlife conflict in different parts of Africa [18].

Finally, the present study results show intense human-livestock predator conflict in the study area. Therefore, the author suggested that conflict mitigation efforts focus on securing the livestock enclosure to protected areas [20]. Mitigation programs and strategies should be designed based on the positive interests of the local people. People who live in and around protected areas suffer a tremendous economic loss due to livestock and crop wildlife damage. Hence, they deserve compensation for losses and ease the economic burden of predator conflict [32, 34]. Ineffective compensation programs may increase the rate of wildlife killings [32, 34, 41]. Parallelly, the concerned government should reduce human settlements, agricultural farmland expansions, and overgrazing in and around the BMNP.

## Conclusion

Human-wildlife conflict has not been a new phenomenon for Ethiopia, where a high level of biological diversity coexists with humans in and around protected areas. Therefore, the findings of this study revealed joint damage from both local people and predators. Hence, the foremost action should be awareness creation about protected areas' environmental, social, and economic importance. Park management is also expected to promote community involvement. It is highly recommended to study the level of damage mainly outside the Park. Rapid action is recommended in appropriate space and time to reduce the long and short-term harm to both wildlife and human beings.

### Some recommendations are provided by the present study

- Park management is expected to promote community involvement.
- Wildlife population assessment is highly recommended outside the Park.
- There should be a buffer zone around the border to reduce conflict.
- Studding population status of olive baboon and spotted hyena.
- Regular compensation fee for farmers that face great damage from wildlife.
- Construct strong pens using stone instead of bamboo or any other soft material would reduce spotted hyena livestock predation.
- However it is hard to apply, redistribution of local people to other suitable territories would certainly reduce wildlife attacks.
- Pastoralist and farmers should enhance their carefulness to reduce livestock loss to some extent.

## Methods

### Study area description

**Fig. 3:**
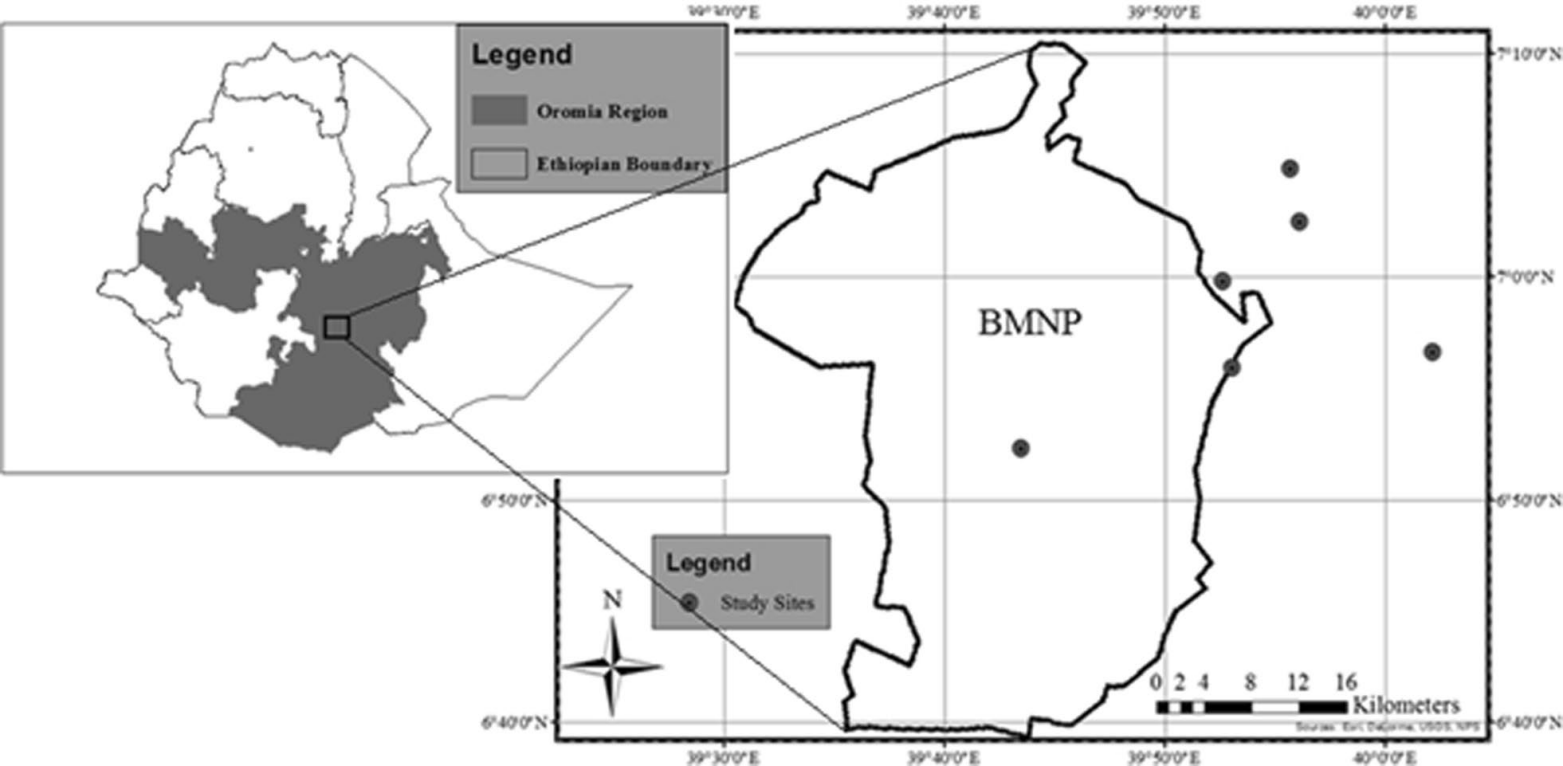
Map of the study area and sample sites in the central and eastern part of Bale Mountains National Park, Ethiopia (This map is produced by the author for the purpose of the present study and other related studies) [16]

Bale Mountains National Park is situated at Bale zone in the Oromia Regional State of Ethiopia. The Park is located within geographical coordinates of 6°29ʹ N–7°10ʹ N and 39°28ʹ E–39°57ʹE (Fig. 3), about 400 km from the capital city of the country. The landscape of the Park is highly varied in size and ranges from 1500 m a.s.l. to 4377 m a.s.l. [49]. Different elevation levels within the Park's boundary determine the temperature, humidity, rainfall amount, and frequency in the area [5, 50]. According to [14], topographical features within the Park can be divided into three categories based upon elevation levels, the northern slopes between 3000 and 3800 m a.s.l. comprised of woodlands, grasslands, and wetlands; the central plateau and peaks from 3800 to 4377 m a.s.l. (Tullu Dimtu) including the second highest mountain point of the country with a central afro-alpine plateau. The southern escarpment of moist tropical forest from 1400 to 3000 m a.s.l. becomes the third topographical category.

Comprising the largest area of Afro-alpine habitat on the continent and the second-largest moist tropical forest in Ethiopia, it harbors few and rare endemic species of the world [45]. Approximately 26% of the Ethiopian endemic faunal community of the Park consists of Ethiopian wolf (Canis simensis), Mountain Nyala (Tragelaphus buxtoni), Menelik’s bushbuck (Tragelaphus scriptus meneliki), Serval (Felis serval), Bohor reedbuck (Redunca redunca), Giant mole rat (Tachyoryctes macrocephalus), Colobus monkeys (Colobus guereza) and many carnivores [1, 2, 27].

## Sampling design and data collection2

### Preliminary survey

A preliminary survey was conducted between December 2018 and February 2019 and information was also gathered about the rough extent of human-predators conflict regarding local people’s perspective. Based on the information gathered during the preliminary study 31 people were interviewed to check the suitability and comprehensibility of the questionnaire according to the local language of the study area.

### Data collection methods

Data were collected from six kebeles (administrative sub-units) from December 2019 to May 2020, using a questionnaire survey of sample households, key informant interviews (elders, Kebele administrators, focal persons, and agricultural and natural resource officers), and field observations. Household questionnaires contained both open and close-ended questions (see Additional file 1). The questionnaire was adapted from the literature [24, 46] and modified for the study (see Additional file 1). Questions concerning every numerical information, general socio-economic status of the community, personal profile, and trend of livestock damage were structured. Open-ended questions about their attitude toward wildlife conservation, predators’ damage controlling methods, causes of human-predators conflict, and their feedback on the problem were semi-structured. Interviewees were selected based on their age, duration of abidance in the study area, and mainly their position in the community [23]. Key informants were interviewed with some open-ended questions designed to gather information about local people’s reactions to livestock predation, how they used and benefited from resources within the Park, and their coexistence with wildlife. Data were presented narratively and compared with individuals’ responses to the questionnaire. Ten years’ retrospective data of livestock damage was collected from sample households and district offices to analyze the monetary loss of households. Photographic field observation (see Additional File 2) was done as a complementary method [25].

### Sample size and sampling technique

In the study sites, a total of 334 households were chosen based on the sample size determination formula of Cochran [7] as follows.

$\mathrm{n}=(\frac{{\mathrm{n}}_{\mathrm{o}}}{1+{\mathrm{n}}_{\mathrm{o}}/\mathrm{N}})$, where n = corrected sample size; n_o_ = required return sample size (based on Cochran’s formula = 384, where margin of error is 0.05); N = total population (2575 households from six Kebeles).

Stratified sampling was adopted to pick sampling units. The study sites were purposively selected based on distance from the Park as to inside the Park (one site), less than 1 km (two sites), from 1 to 5 km, and greater than 5 km (three sites). Households were selected randomly from each kebele. Respondents from each household were randomly selected for interview on a first-come, first-served basis. Respondents were randomly selected with some alteration of sex [33]. All interviews of 334 households were conducted by 12 data collectors with the aid of field assistants who were selected from the community based on their experience of abode in the study sites. Participant members of the community and their local leaders were informed about the aim of the study and asked for proceeding permission [26] and verbal consent was obtained from all respondents.

### Data analysis

Data were analyzed using descriptive statistics and responses were compared with the chi-square test. A correlation was also done to determine the relationship between livestock damage and distance from the Park.

## Supplementary Information

**Additional file 1. **Questionnaires. The data gathering questionnaires were developed after a critical and detailed reviews of previously conducted similar research.

**Additional file 2.** Photographs. In this file, photographs of injured and killed livestock are included. Furthermore, local communities have been expanding their activities in different forms. Some of the activities were captured and included in the file to evidence the results of the present study. Additional photographs that were obtained from the Park’s administration database are also included.

## Data Availability

The data used and analyzed during the current study is available from the corresponding author on a reasonable request, without disclosure of the interviewees.

## Abbreviations

a.s.l: Above sea level
BMNP: Bale Mountains National Park; Kebeles (AT-Aloshe Tilo, WT-Waltai Tosha, WA-Waltai Azira, FA-Fassil Angesso, IS-Ititu Sura)
