# Supplementary material for: Humans-livestock predators conflict in the Central and Eastern Part of Bale Mountains National Park, Ethiopia
Source: BMC Ecol Evol. 2022 Oct 4;22:113. doi: 10.1186/s12862-022-02065-y (PMC9531451; doi:10.1186/s12862-022-02065-y)
Supplement: Supplementary file 1 — Additional file 1. Questionnaires. The data gathering questionnaires were developed after a critical and detailed reviews of previously conducted similar research. [file 12862_2022_2065_MOESM1_ESM.docx]

**Additional files - Questionnaires**

1. **Introductory questions**
   1. Code­­­­­­­­­­­­­_____________________
   2. Age ____________________
   3. Sex ____________________
   4. Residence (Kebele) _____________________
   5. Estimated distance from the study area ______________
   6. Family size _________________
   7. Education level (illiterate, informal education, primary, Secondary, beyond secondary education)_____________
2. **Principal questions**
   1. The size of farmland__________, Grazing land____________, woodlot_________
   2. The type of crop do you grow

__________ ______________ ___________

___________ ______________ ___________

- 1. How much did you get last year? __________
  2. The type and number of livestock you have.

**Type no**.

1. ________________ ____
2. ________________ ____
3. ________________ ____

­­­­­­­­­

- 1. What are your livelihood activities?

1. Crop production ___________ b. Livestock keeping ______c. Farming and livestock keeping ___________ d. Trade ______e. Other (mention) _________
   1. Where does your livestock graze?

A. in the study area_________ B. outside the study area_________ C. in a private grazing land (if they possess)

- 1. How long do they graze in the study area?

A. 1-3 months_________ C. 6-9 months___________

B. 3-6 months_________ D. 9-12 months__________

2.8. List the type of resources you and your livestock use from the Park?

___________ ______________ ______________

___________ ______________ ______________

- 1. List the type of wild animals that you know in the BMNP

___________ ______________ ________________

___________ ______________ ________________

___________ ______________ ________________

- 1. Do wild animals cause damage to your crops and livestock?

A. Yes B. No

2.11. List wild animals that have been causing damages, and type and extent of damage?

| **No** | **Animal type** | **Type of damaged** | **Extent of damage** |
| --- | --- | --- | --- |
|  |  |  |  |
|  |  |  |  |
|  |  |  |  |
|  |  |  |  |

- 1. What are the different techniques used to control (reduce) the damage (including crop damage) caused by these animals?

1. ____________________________________
2. ____________________________________
3. ___________________________________
   1. Were your techniques successful to reduce the extent and tendency of damage?
4. Yes B. No
   1. What and where do you collect your firewood?

A. from the park B. outside the park

- 1. List the type of wild animals depredates livestock?

______________ _____________ ________________

______________ _____________ ________________

- 1. Have you ever seen predator or baboons taking sheep, goat, calves and others?

(If yes) mention type of predator and livestock.

| **No** | **Predator** | **Type of**  **livestock**  **predated** | **Number killed within** | | | **Growth stage of livestock** |
| --- | --- | --- | --- | --- | --- | --- |
|  |  |  | Last 10 years | Last five years | last year |  |
|  |  |  |  |  |  |  |
|  |  |  |  |  |  |  |
|  |  |  |  |  |  |  |

- 1. How do you minimize the killing of livestock by the predators?

1. _______________________________
2. _______________________________
3. _______________________________
   1. Do you have dogs?
4. Yes B. No
   1. Was there any Rabies incidence in last 5 years?
   2. How do you feel about conserving these conflict causing animals?

______________________________________________________________

- 1. Do you think conserving wildlife is important?

______________________________________________________________

- 1. What measures do you think should be taken by the following in order to prevent the crop damage?

1. By the government ___________________________________________
2. By the private sector ____________________________________________
3. By the farmer ___________________________________________

**Appendix II- Focus group Discussion Questions**

1. Do you think the presence of the BMNP close to your area benefited the community?

2. What benefits have been realized up until now?

3. Do you think local people and their livestock affect wildlife?

4. How do local community and wildlife in the BMNP could coexist in peace and harmony?

5. What is your attitude towards conflict causing animals?

6. What is the importance of conserving wildlife?
