# Supplementary material for: Humans-livestock predators conflict in the Central and Eastern Part of Bale Mountains National Park, Ethiopia
Source: BMC Ecol Evol. 2022 Oct 4;22:113. doi: 10.1186/s12862-022-02065-y (PMC9531451; doi:10.1186/s12862-022-02065-y)

Additional files 2 - Photographic information

Photo 1. Local people do alternative things while watching livestock predators (this picture was taken by the author* for the purpose of this study).


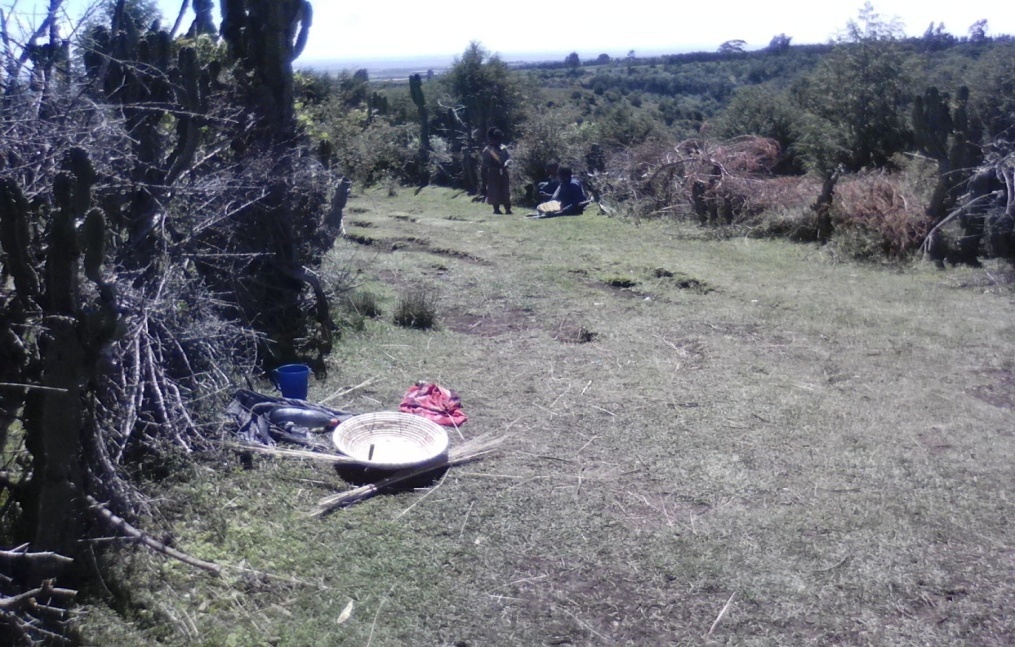


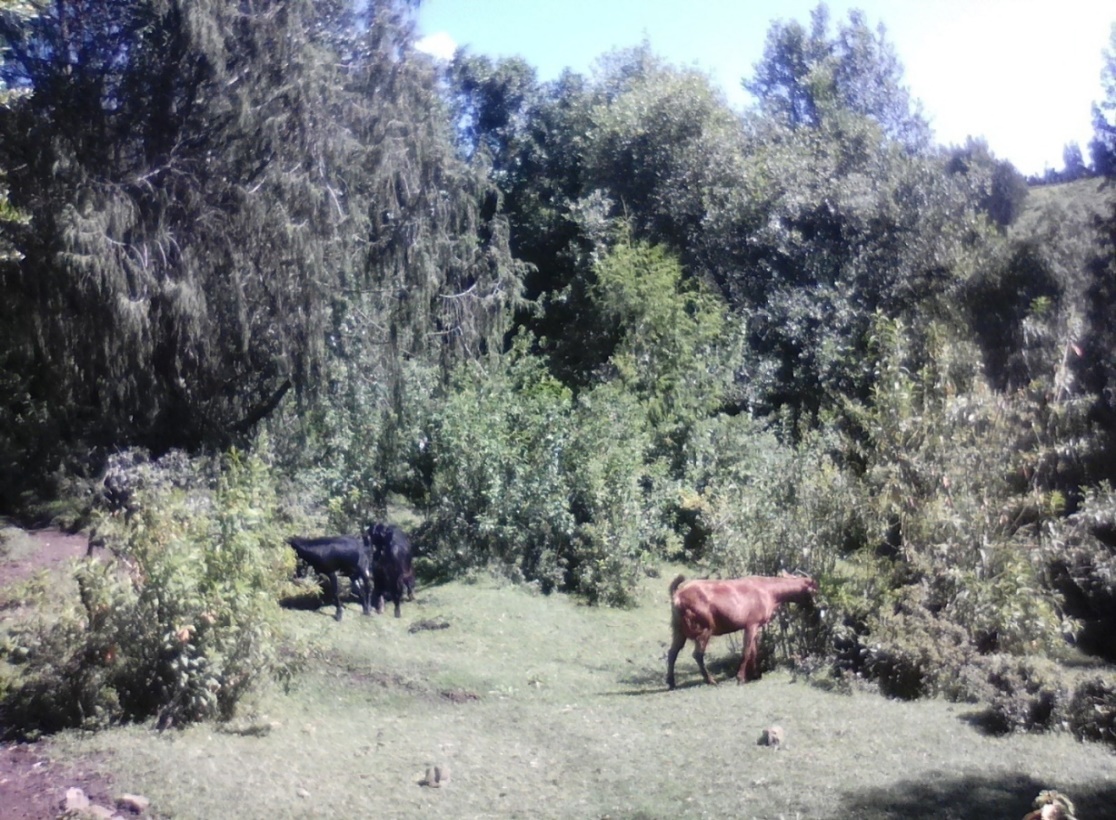


Photo 2. A horse predated by spotted hyena at night outside home as evidenced by its holders, 2020 G.C. (this picture was taken by the author* for the purpose of this study).


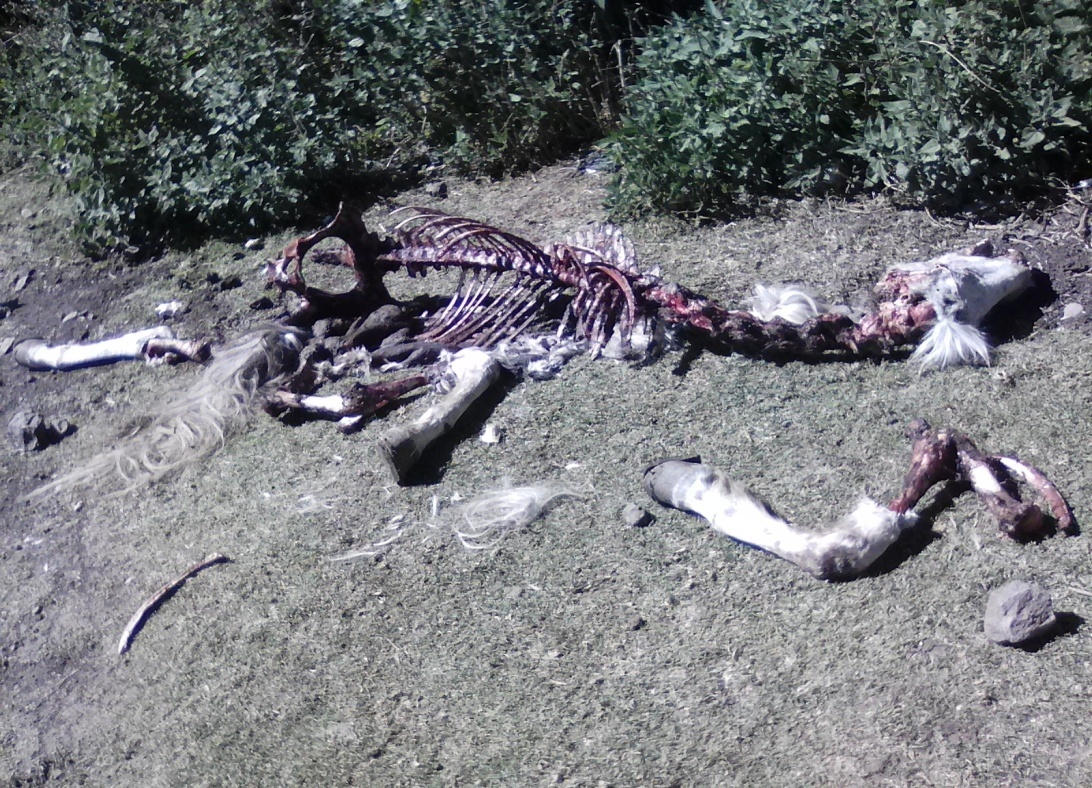


Photo 3. A goat with injured left eye by olive baboon, 2019 G.C. (this picture was taken by the author* for the purpose of this study).


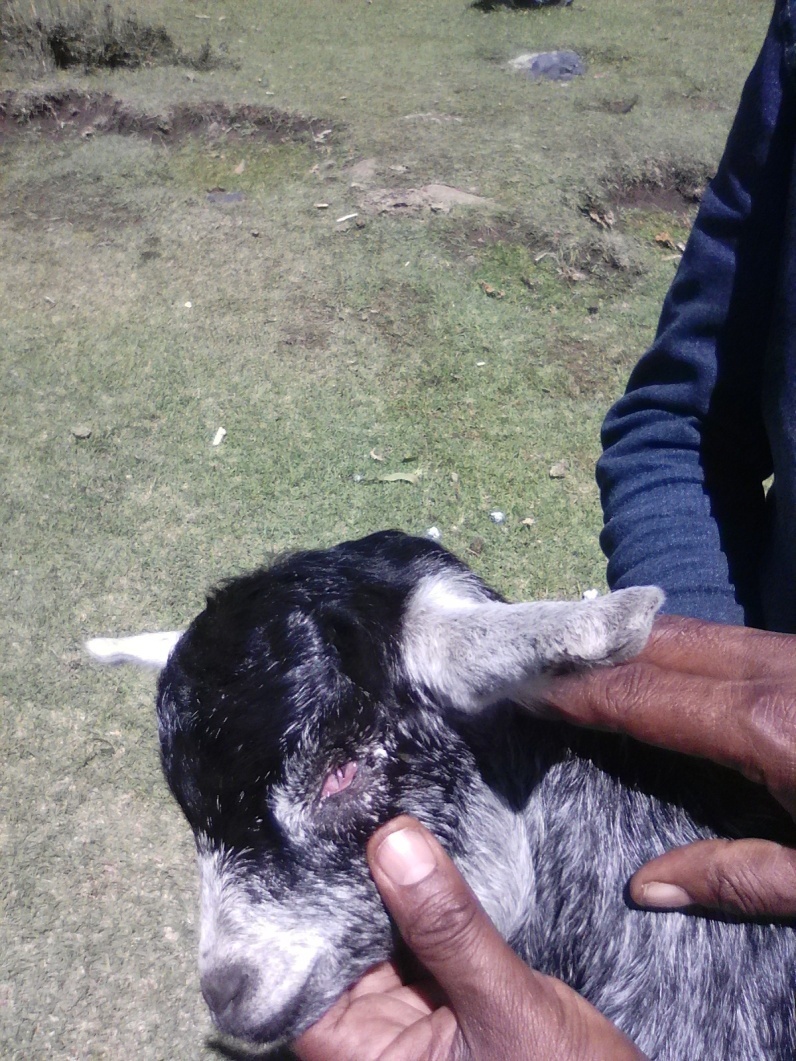

Supplement: Supplementary file 2 — Additional file 2. Photographs. In this file, photographs of injured and killed livestock are included. Furthermore, local communities have been expanding their activities in different forms. Some of the activities were captured and included in the file to evidence the results of the present study. Additional photographs that were obtained from the Park’s administration database are also included. [file 12862_2022_2065_MOESM2_ESM.docx]
